# A quantitative definition of scaphoid union: determining the inter-rater reliability of two techniques

**DOI:** 10.1186/1749-799X-8-28

**Published:** 2013-08-21

**Authors:** Ruby Grewal, Uri Frakash, Said Osman, Robert Y McMurtry

**Affiliations:** 1 Department of Surgery, Division of Orthopedic Surgery, Hand and Upper Limb Center, St. Joseph's Health Care, University of Western Ontario, 268 Grosvenor St, London, Ontario N6A 4L6, Canada; 2 Department of Medical Imaging, St. Joseph's Health Care, University of Western Ontario, 268 Grosvenor St, London, Ontario N6A 4L6, Canada

**Keywords:** Inter-rater reliability, Partial union, Scaphoid, Union

## Abstract

**Background:**

Despite extensive literature supporting the use of computerized tomography (CT) scans in evaluating scaphoid fractures, there has not been a consensus on the methodology for defining and quantifying union. The purpose of this study was to test the inter-observer reliability of two methods of quantifying scaphoid union.

**Methods:**

The CT scans of 50 non-operatively treated scaphoid fractures were reviewed by four blinded observers. Each was asked to classify union into one of three categories, united, partially united, or tenuously united, based on their general impression. Each reviewer then carefully analyzed each CT slice and quantified union based on two methods, the mean percentage union and the weighted mean percentage union. The estimated percentage of scaphoid union for each scan was recorded, and inter-observer reliability for both methods was assessed using a Bland-Altman plot to calculate the 95% limits of agreement. Kappa statistic was used to measure the degree of agreement for the categorical assessment of union.

**Results:**

There was very little difference in the percentage of union calculated between the two methods (mean difference between the two methods was 1.2 ± 4.1%), with each reviewer demonstrating excellent agreement between the two methods based on the Bland-Altman plot. The kappa score indicated very good agreement (Ƙ = 0.80) between the consultant hand surgeon and the musculoskeletal radiologist, and good agreement (Ƙ = 0.62) between the consultant hand surgeon and the hand fellow for the categorical assessment of union.

**Conclusions:**

This study describes two methods of quantifying and defining scaphoid union, both with a high inter-rater reliability. This indicates that either method can be reliably used, making it an important tool for both for clinical use and research purposes in future studies of scaphoid fractures, particularly those which are using union or time to union as their endpoint.

**Level of evidence:**

Diagnostic, level III

## Background

Scaphoid union, as determined by X-ray, is the designated end point for most comparative trials evaluating treatment effectiveness in the study of scaphoid fractures. However, it has been shown by several authors that plain radiography is not a reliable method of assessing scaphoid fractures [1-3]. Despite this, plain radiographs form the primary basis of evaluating union in most published level one trials comparing ORIF to casting of acute scaphoid fractures [4-9]. A standardized, reliable, valid method of defining scaphoid union is needed before our understanding of scaphoid fractures can be improved and the literature can be critically evaluated.

Modalities used to assess scaphoid union have been studied by several authors. Dias et al. demonstrated that there was poor agreement between observers on whether trabeculae crossed the fracture line and concluded that inter-observer agreement on scaphoid union based on plain radiographs was poor [1]. Dias et al. also reported that radiographs taken 12 weeks after a scaphoid fracture do not provide reliable and reproducible evidence of healing [1]. Many authors have reported that computerized tomography is the preferred method of assessing scaphoid fracture location, deformity, displacement, and union status [2,10-14], and it was also reported by Bain that computerized tomography (CT) scans have a higher correlation with operative findings [14].

Despite the body of literature supporting the use of CT scans to evaluate scaphoid fractures, there has not been a consensus on the methodology for defining and quantifying union. Singh et al. describe a method for quantifying the percentage of scaphoid union based on CT scan in 2005 [13]. By describing the percentage of united bone seen on a given CT scan, scaphoid healing can be quantified at a certain point along the spectrum of union and discussed not only as united or un-united but also partially united. The phenomenon of partial union has been described by several authors [2,3,13] and is important to be recognized in order to avoid misdiagnosing a partially united fracture as un-united based on X-ray only to find intra-operatively that it appears healed, as has been described in the literature [1,2].

In our center, we routinely use CT scans to determine the percentage of union of the scaphoid in a method similar to that described by Singh et al. [13]. Our treatment decisions are based on the percentage of united bone and the surgeon's clinical assessment.

The reliability of the method used to determine percentage of union has not been examined. The purpose of this study was to determine a method of reliably quantifying scaphoid union on CT scan and to examine the inter-observer reliability of this technique.

## Methods

A radiology database at a tertiary care upper extremity center was searched for all scaphoid CT scans performed from 2004 to 2010 inclusive. Fifty acute scaphoid fractures were selected and extracted from this database. Only non-operatively treated scaphoid fractures were included in this study, and cases were excluded if patients were skeletally immature. Each case was reviewed by an independent evaluator to determine eligibility for inclusion in this study. The time between CT scan and injury was not standardized. The independent evaluator was instructed to conduct a random sampling to ensure that time from injury would not influence results and a wide range of united to un-united fractures would be included. The mean time interval between injury and scan ranged from 1 to 197 days with a mean of 64.5 ± 46.8 days. This independent evaluator also reviewed the radiology reports to ensure that the 50 selected scans represented a range of united, partially united, and tenuously united cases. The reviewers were blinded to all case identifiers and to the results of their co-investigators.

All scaphoids were scanned using a CT scanning technique previously reported [15], using 0.625-mm helical cuts through the long axis of the scaphoid. The images were reviewed using the GE Centricity PACS RA 1000 system (GE Healthcare, Fairview, CT, USA), with the sagittal cuts being used to assess fracture healing. Each scan was assessed by four observers: two consultant hand surgeons, a musculoskeletal radiologist, and a hand surgery fellow.

**Figure 1:**
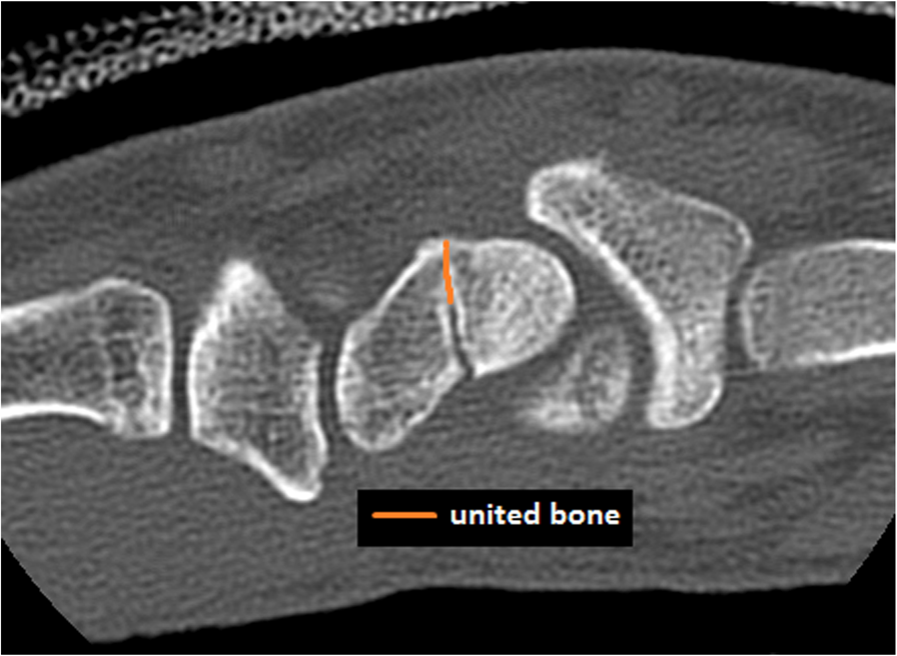
Length of the fracture line which is united.

**Figure 2:**
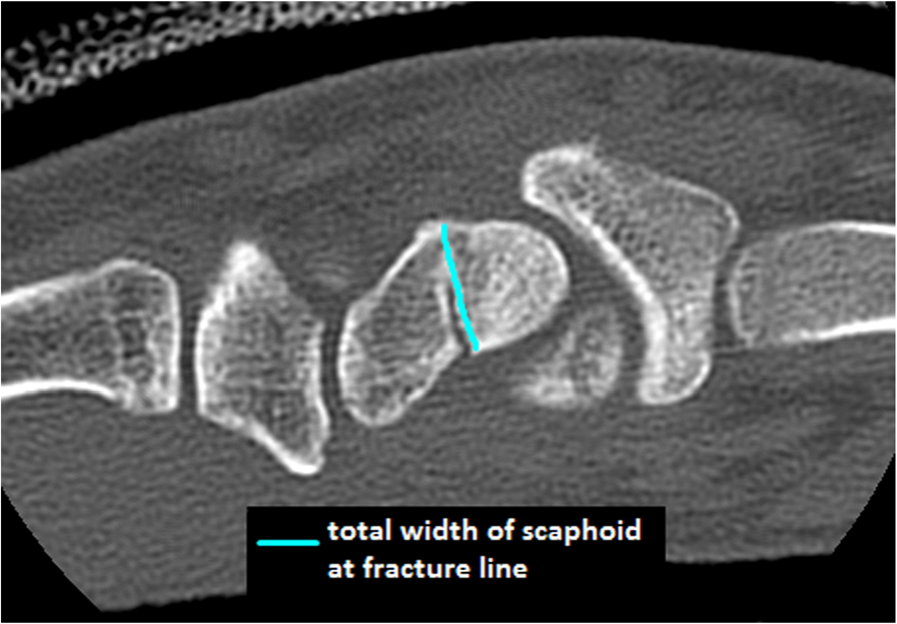
Total width of scaphoid at the fracture line.

Each of the four reviewers was blinded to the clinical details of the case and blinded to the results of the other reviewers. Each reviewer was first asked to identify the location of the fracture (distal pole, waist, or proximal pole). Based on their general impression of the fracture, the reviewer was then asked to classify union into one of three categories, united (75%–100%), partially united (50%–75%), or tenuously united (≤50%). Each reviewer then aimed to quantify union by carefully analyzing each CT slice containing the fracture line and adjacent scaphoid. The fracture line was evaluated and the length of united bone (Figure 1) was compared to the total width of the scaphoid at the fracture line (Figure 2). These two measurements were then used to quantify union based on the two following methods.

### Mean percentage union

The method used to quantify mean percentage union was based on that described by Singh et al. [13]. The length of united bone (Figure 1) along the fracture line was measured and divided by the total width of the scaphoid (Figure 2), giving a percentage of union for the slice.

$$\begin{array}{l} \mathrm{Percentage}\;\mathrm{of}\;\mathrm{union}=\frac{\mathrm{Sum}\;\mathrm{of}\;\mathrm{the}\;\mathrm{total}\;\mathrm{length}\;\mathrm{of}\;\mathrm{united}\;\mathrm{scaphoid}\;\left(\mathrm{mm}\right)}{\mathrm{Sum}\;\mathrm{of}\;\mathrm{the}\;\mathrm{total}\;\mathrm{length}\;\mathrm{of}\;\mathrm{fracture}\;\left(\mathrm{mm}\right)}\\ \;\;\;\times \;100\% \end{array}$$

The percentage of union for each slice was then averaged to obtain a value for the overall percentage of united scaphoid. Using this technique, each CT slice was weighted equally. Peripheral slices involving a small amount of bone were weighted equally to the larger central slices involving more bone. As a result, there was a theoretical concern that this method may not provide an accurate estimation of overall union as slices in the periphery (whether united or un-united) would cause the overall mean percentage calculation to be skewed. As a result, we sought to devise and test a second method which we termed the weighted mean percentage union.

### Weighted mean percentage union

In order to weight slices involving a greater portion of the scaphoid more heavily, we calculated the overall percentage of scaphoid union based on the total millimeters of bone involved. We divided the sum of the millimeters of united bone on all cuts by the sum of the fracture width on all slices.

For example, the width of the scaphoid at the fracture line may be 10 mm on one slice, but only 4 mm at the other. If one slice shows that 2 mm is united over a total fracture width of 4 mm and the other slice shows that all 10 mm of the fracture is united, the weighted mean percentage of union would be calculated as follows:

$$\begin{array}{l} \mathrm{Percentage}\;\mathrm{of}\;\mathrm{union}\;=\;\frac{2\;\mathrm{mm}+10\;\mathrm{mm}}{4\;\mathrm{mm}+10\;\mathrm{mm}}\;\\ \;\;\;=\;\frac{12}{14}\;\times \;100\%\;\\ \;\;\;=\;85.7\%. \end{array}$$

Based on the method of Singh et al. [13], this would have been calculated as a mean 75% union based on the average of one slice which is 50% united (2 mm/4 mm) and the other slice which is 100% united (10 mm/10 mm).

We tested the reliability of both methods and also determined if the assessment of percentage of union changed significantly based on the two different calculations.

### Statistics

Using power curves generated by Donner and Eliasziw [16], with four raters, it was determined that a sample size of 45 was needed to ensure 80% power with alpha 0.05. Statistical analysis was performed with the use of SPSS statistical package (version 17.0; SPSS, Chicago, IL, USA). The estimated percentage of scaphoid union for each scan (by both methods) was recorded, and inter-observer reliability was assessed using a Bland-Altman plot to calculate for the 95% limits of agreement. The Bland-Altman plot was used to determine the degree of agreement between the percentage of union obtained by each of the two methods described (mean percentage and weighted mean percentage) for all reviewers. Kappa statistic was used to measure the degree of agreement for the categorical assessment of union (united, partially united, and tenuously united) among raters. The kappa statistic was interpreted according to the definitions of Landis and Koch where <0.2 indicated poor agreement, 0.2–0.4 fair, 0.4–0.6 moderate, 0.6–0.8 good, and 0.8–1.0 very good agreement [17].

## Results

**Figure 3:**
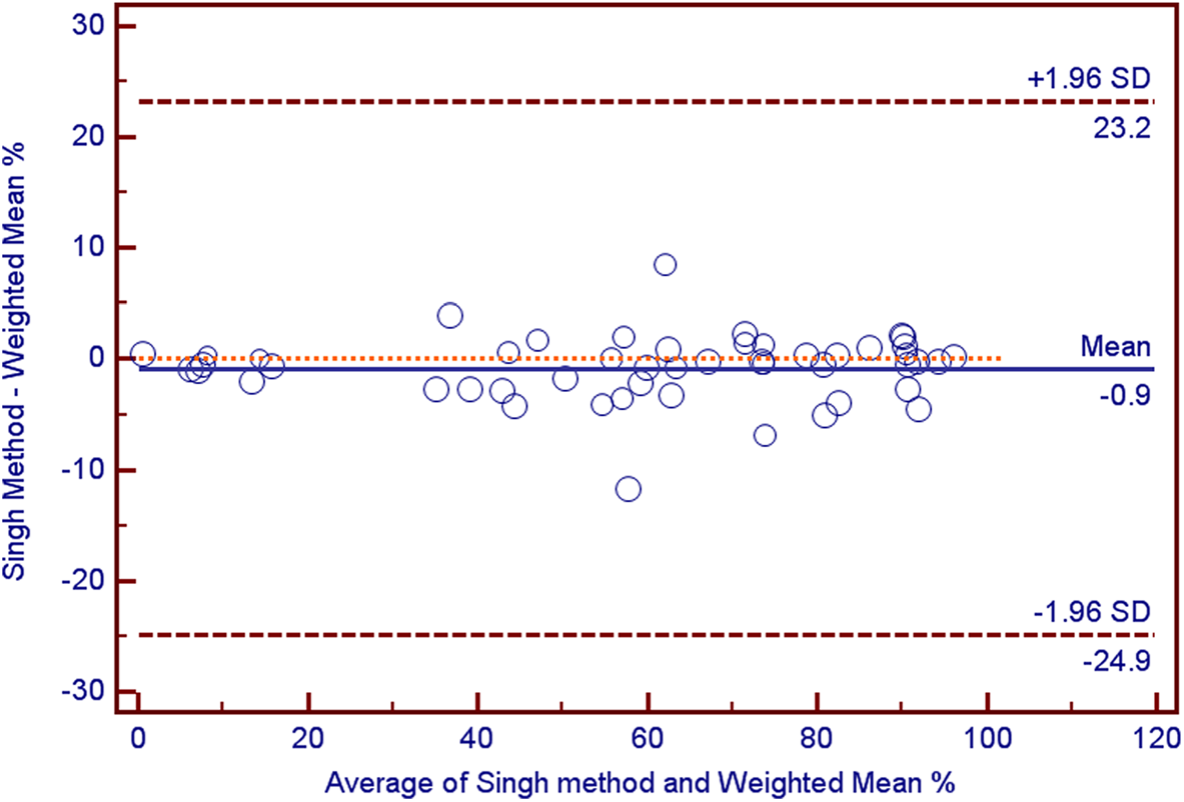
Bland-Altman plot comparing two methods of determining percentage of union.

Each reviewer demonstrated excellent agreement between the values for percentage of scaphoid union obtained by each of the two methods. The mean difference calculated between the two methods was minimal at 1.2 ± 4.1%. This is depicted in the Bland-Altman plot (Figure 3) which indicates that both methods have excellent agreement. There did not seem to be a significant variation in agreement depending on the percentage of cross section which has united; however, there was a slightly greater agreement on cases with very little union (i.e., <10%).

**Figure 4:**
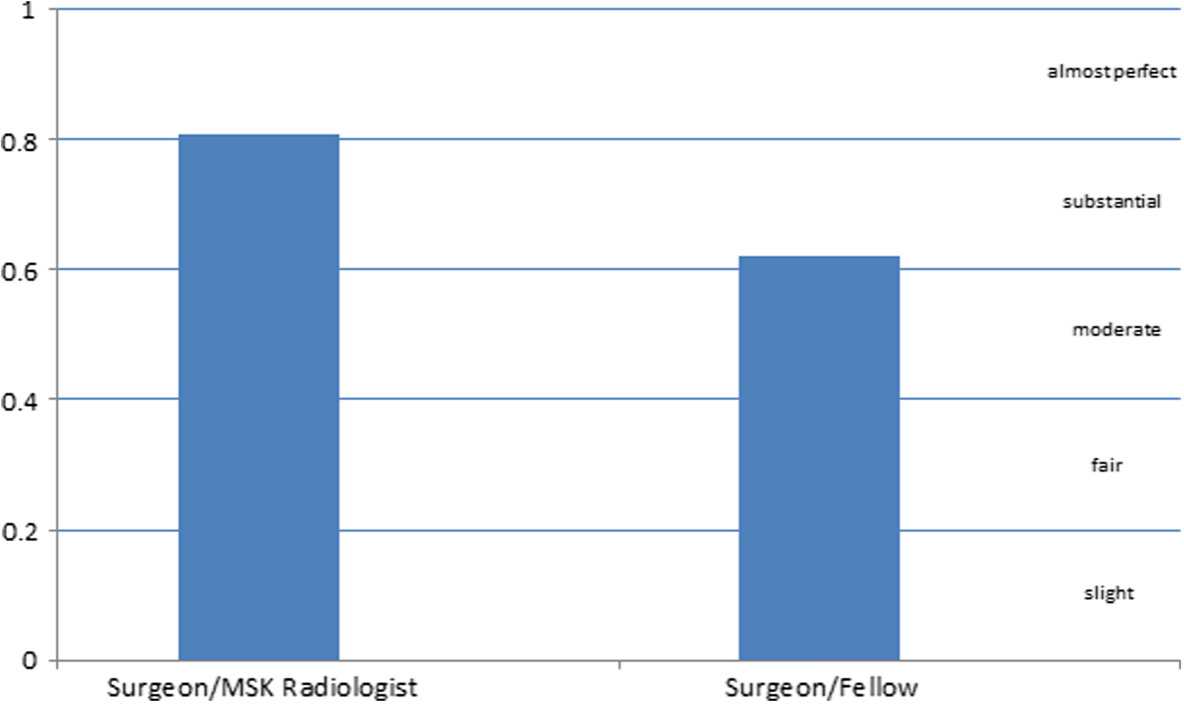
**Kappa scores for the measure of agreement for the categorical assessment of union.** United, partially united, and tenuously united.

The kappa score indicated very good agreement (Ƙ = 0.80, 95% confidence interval 0.65–0.93, *p* < 0.001) between the consultant hand surgeon and the musculoskeletal radiologist, and good agreement (Ƙ = 0.62, 95% confidence interval 0.44–0.80, *p* < 0.001) between the consultant hand surgeon and the hand fellow (Figure 4).

## Discussion

This study has described two different methods of quantifying scaphoid union and has shown that there is excellent inter-rater reliability with both techniques. Singh et al. also demonstrated high inter-observer agreement between two judges when rating the CT scans of scaphoid fractures as united, un-united, or unsure (kappa 0.77) [13]. This is the first study to demonstrate excellent reliability of the calculated percentage of scaphoid union among four observers and also a high inter-observer agreement (0.8) between experienced judges rating scaphoid fractures as united, partially united, or tenuously united. This method can be used for both surgical and non-surgically treated cases; however, the hardware will obscure the images and make interpretation more difficult. While we use this method clinically in surgical cases, this study was not able to establish the reliability of these methods in this setting.

Although the method of determining the weighted mean percentage can be tedious and cumbersome in a busy clinical practice, the method of determining the mean percentage [13] produces comparable results and does not skew the final calculation in any significant way. We routinely use the method of Singh et al. [13] to quantify the extent of union. With practice and experience, we have found the method to be simple and straightforward to implement.

Because plain radiographs cannot reliably confirm union [3,13], many scaphoid fractures are likely immobilized longer than necessary. Geoghegan et al. report success following 4 weeks of immobilization for undisplaced scaphoid waist fractures that have evidence of union or partial union on the CT scan at week 4 [18]. When clinical decisions are based on X-rays only, this may lead to prolonged, unnecessary immobilization or an unnecessary push towards surgery [18]. By determining the percentage of union for scaphoid fractures based on CT scan, we can more accurately follow these fractures as they progress through the phases of healing. The clinical implications of this measurement are not clear, and further studies will be needed to determine when union is sufficient enough to allow treatment progression (i.e., discontinuation of immobilization, rehabilitation, return to work/sport).

## Conclusions

This study describes two methods of quantifying and defining scaphoid union, both with a high inter-rater reliability. This indicates that either method can be reliably used, making it an important tool for both clinical use and research purposes in future studies of scaphoid fractures, particularly those which are using union or time to union as their endpoint.

## Competing interests

The authors declare that they have no competing interests.

## Authors’ contributions

RG conceived of the design of the study. RG, UF, SO, and RYM read and interpreted the scaphoid CT scans images, and contributed to the design of the study. RG prepared the manuscript. RYM, SO, and UF edited the manuscript. All authors read and approved the final manuscript.
